# Supplementary material for: SCING: Inference of robust, interpretable gene regulatory networks from single cell and spatial transcriptomics
Source: iScience. 2023 Jun 14;26(7):107124. doi: 10.1016/j.isci.2023.107124 (PMC10331489; doi:10.1016/j.isci.2023.107124)
Supplement: Document S1. Figures S1–S17 [file mmc1.pdf]

iScience, Volume *26*

Supplemental information

SCING: Inference of robust, interpretable gene regulatory networks from single cell and spatial transcriptomics

Russell Littman, Michael Cheng, Ning Wang, Chao Peng, and Xia Yang

Supplemental Information

SCING: Inference of Robust, Interpretable Gene Regulatory Networks from Single Cell and Spatial Transcriptomics

Russell Littman, Michael Cheng, Ning Wang, Chao Peng & Xia Yang

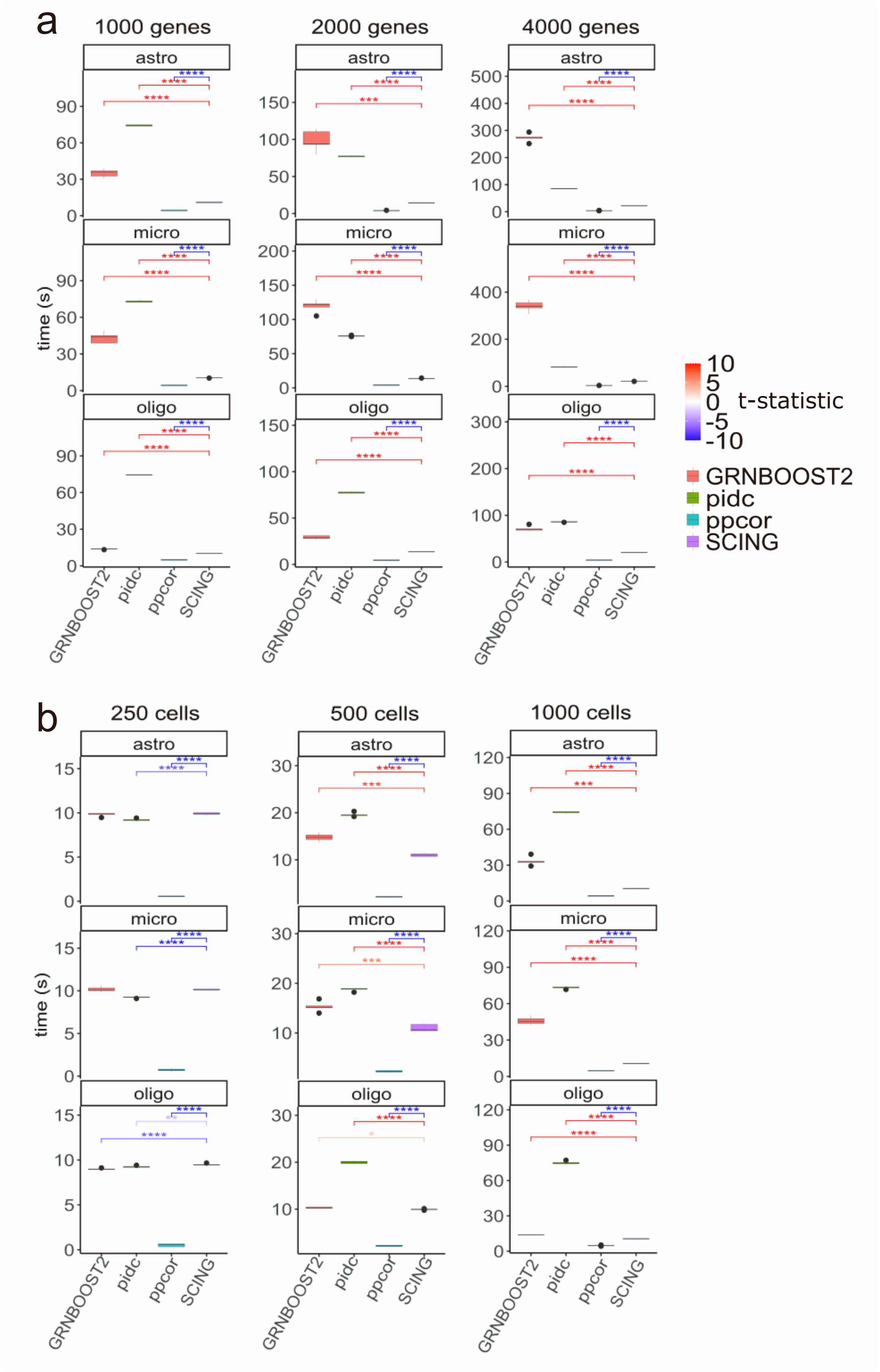

Figure S1. Run time comparison of methods for building a single GRN, related to Figure 1. (a) SCING is faster than GRNBOOST2 and PIDC as the number of genes increases. The test increasing the number of genes was performed on 1000 cells. (b) SCING is faster than GRNBOOST2 and PIDC as the number of cells increases. The test increasing the number of cells was performed on 1000 genes. ppcor is faster than all other methods. Red significance indicates that SCING is faster, while blue indicates SCING is slower.
(*: $p < 0.05$, **: $p < 0.01$, ***: $p < 0.001$, ****: $p < 0.0001$)

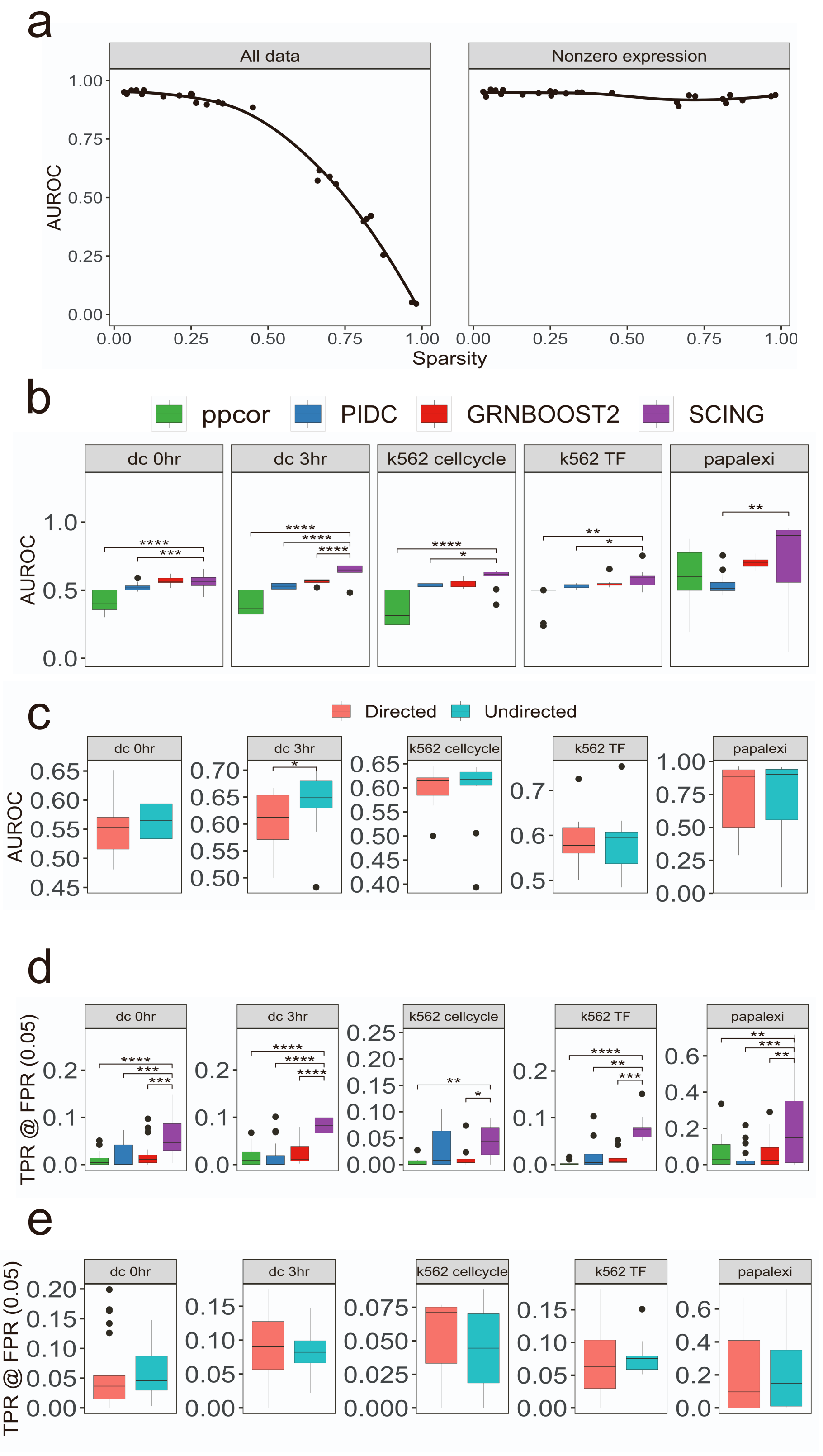

Figure S2. Predicted downstream affected genes of perturb-seq based perturbation in 5 datasets with GRNs built on all cells in each dataset, related to Figure 2. (a) Performance of SCING in predicting downstream gene perturbation as a function of the fraction of zeros in each perturbation, before and after removing cells with zero expression of the gene of interest. (b) Area under receiver operator characteristic (AUROC) curve for prediction of downstream perturbations using undirected GRNs. (c) AUROC for prediction of downstream perturbation on directed GRNs for SCING. (d) True positive rate (TPR) at a false discovery rate (FDR) of 0.05 for the prediction of downstream perturbations on undirected GRNs. (e) TPR at FDR of 0.05 for the prediction of downstream perturbations on directed GRNs for SCING.
(*: $p < 0.05$, **: $p < 0.01$, ***: $p < 0.001$, ****: $p < 0.0001$)

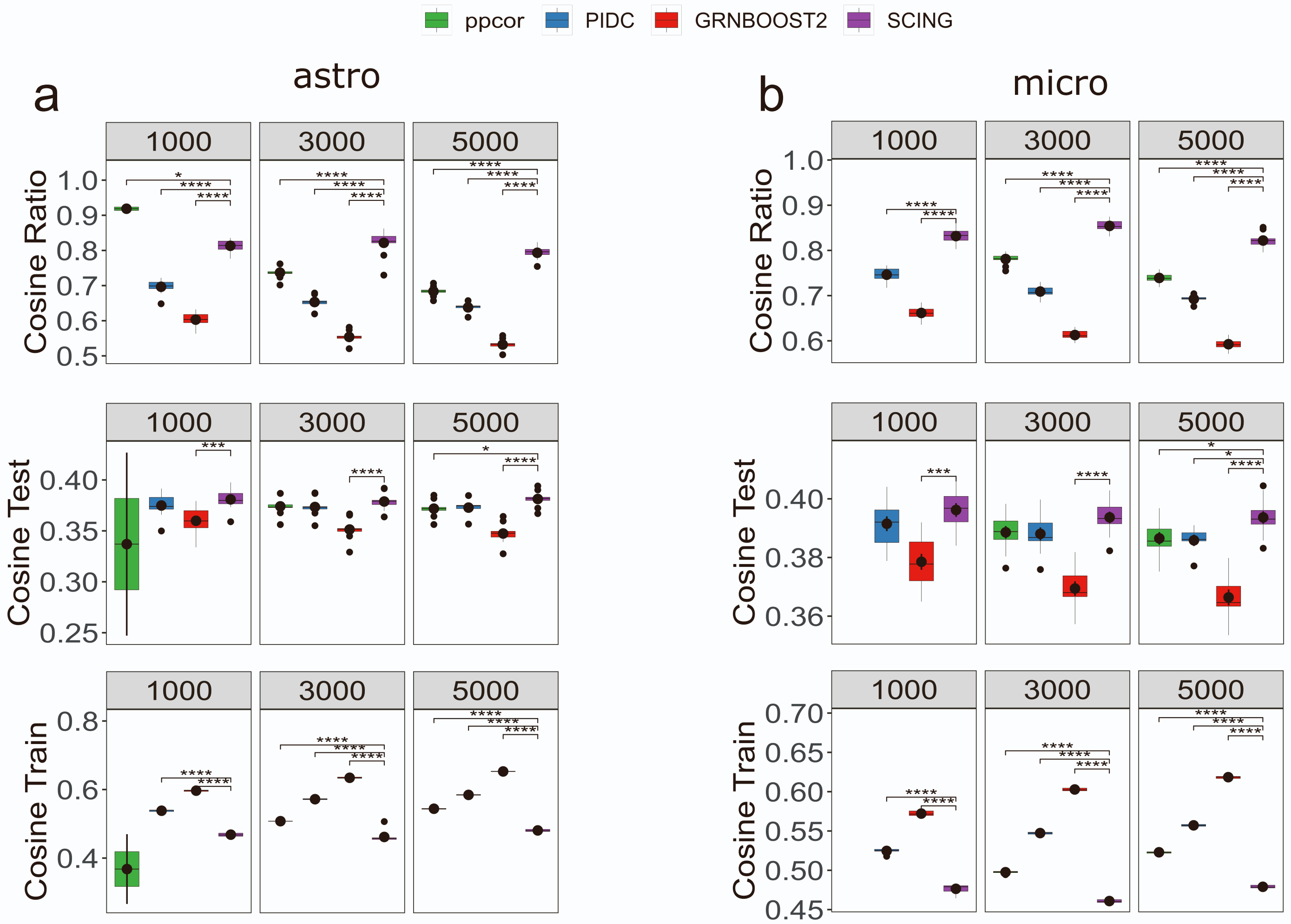

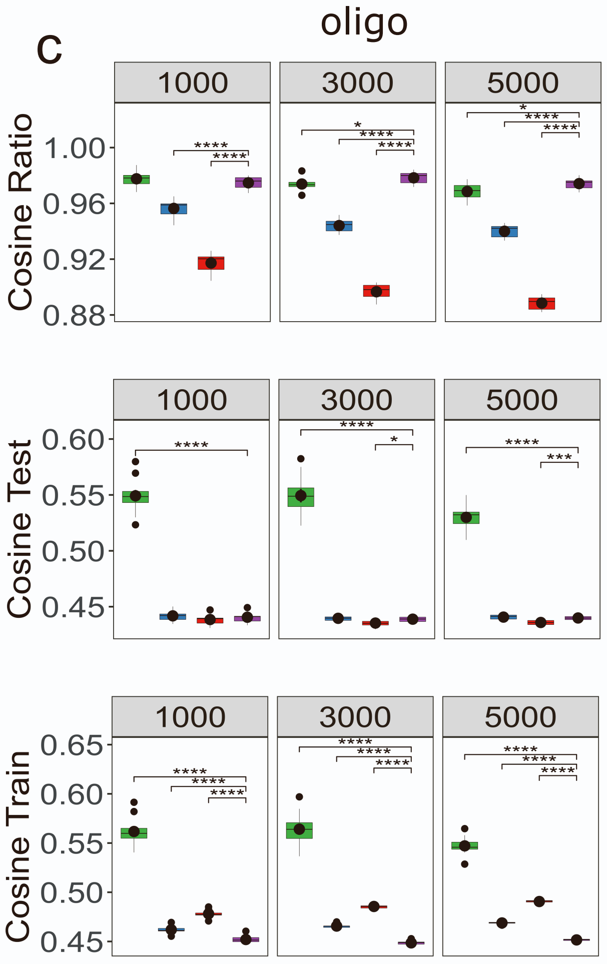

Figure S3. Robustness of GRN inference methods across different numbers of genes in astrocytes, microglia, and oligodendrocytes, related to Figure 2. We tested the cosine similarity metrics between held out testing cells and the predicted expression of each gene for astrocytes (a), microglia (b), and oligodendrocytes (c) in the Morabito et al. dataset. This was done on 1,000, 3000, and 5,000 genes for 10 subsamples each. The gradient boosting regressor was trained on training cells based on each method's GRN output. We note that ppcor could not find any significant edges for the 1,000 gene tests for the microglia. (a, b, and c) In most test cases, SCING outperforms other methods in the cosine ratio (overfitting metric), is similar to or outperforms methods in cosine similarity score in the test data (cosine test), showing the SCING network is just as informative as other approaches without overfitting the training data. This result is mostly consistent across different numbers of genes.

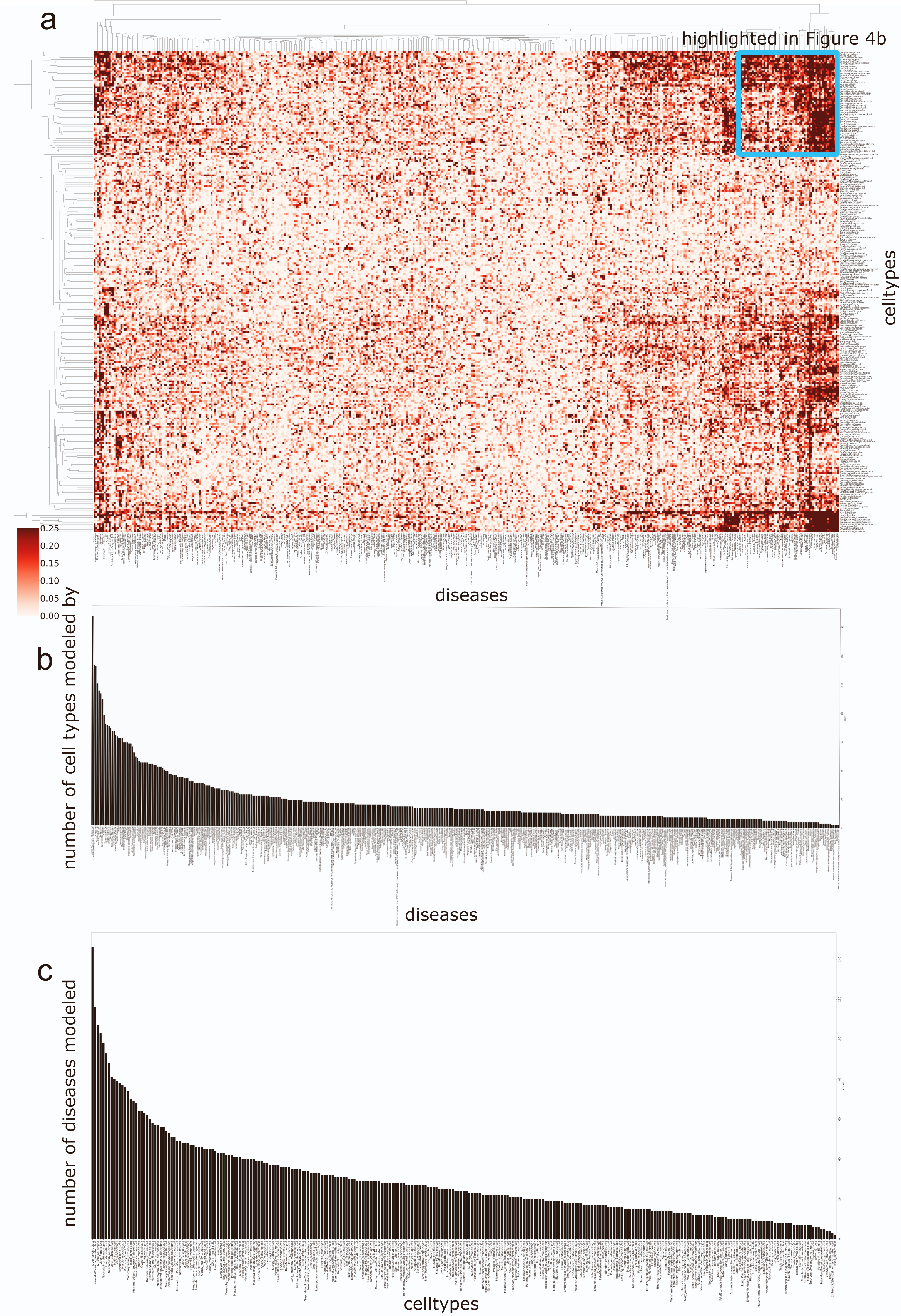
a
highlighted in Figure 4b
celltypes
diseases
0.25
0.20
0.15
0.10
0.05
0.00
b
number of cell types modeled by
diseases
c
number of diseases modeled
celltypes

Figure S4. SCING GRNs' modeling capabilities of DisGeNET diseases on entire MCA, related to Figure 4. (a) Heat map of all cell types in mouse cell atlas and SCING GRNs modeling of disease subnetworks across all diseases in DisGeNET. (b) Number of cell types each disease subnetwork can be accurately (>0.1) modeled by. (c) Number of disease subnetworks accurately modeled (> 0.1) by each cell type.

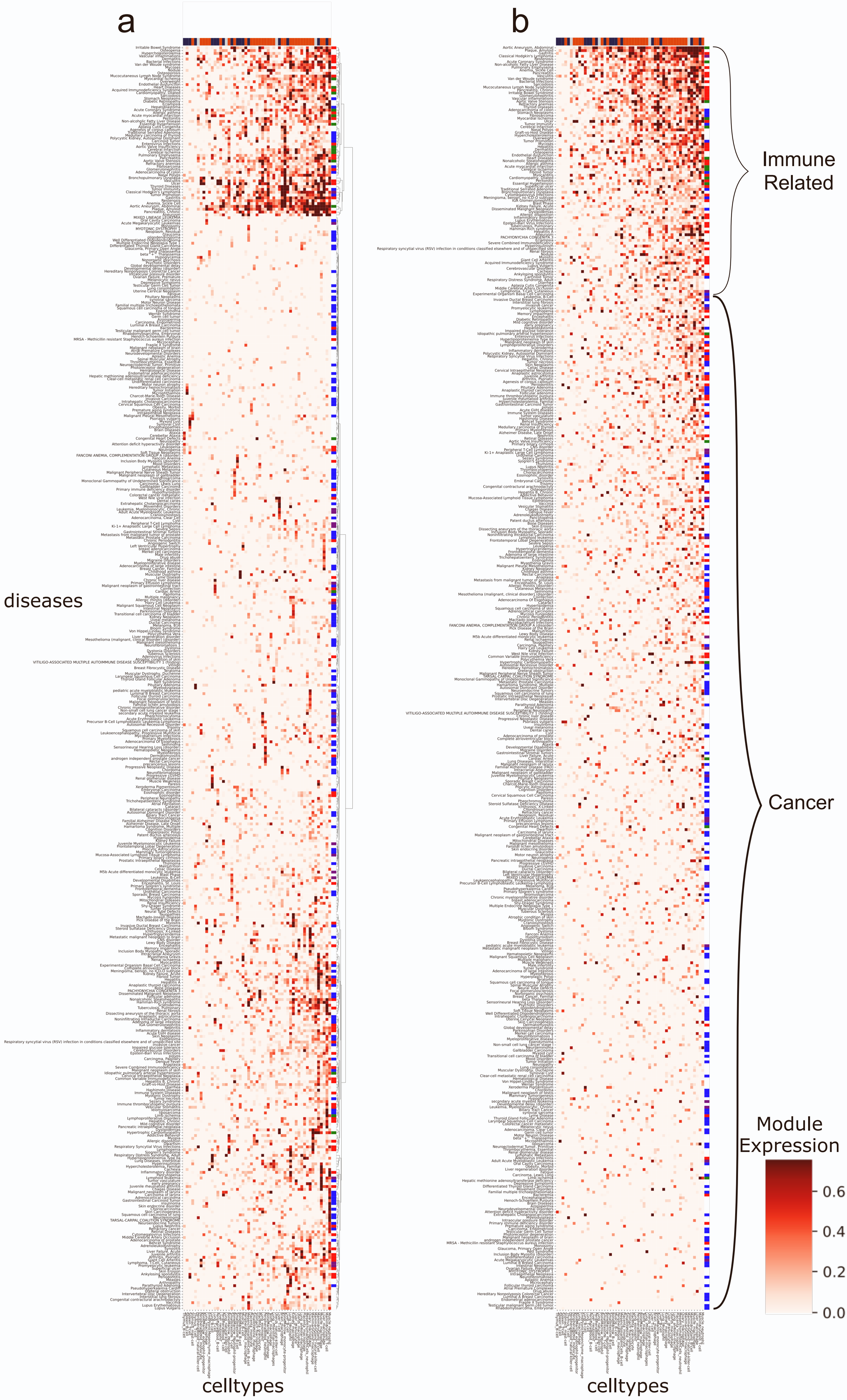
a
b
diseases
celltypes
celltypes
Immune Related
Cancer
Module Expression
0.6
0.4
0.2
0.0

Figure S5. Clustermap of immune cell types and all diseases, related to Figure 4. (a) Cell types are ordered by the number of diseases they accurately model, and diseases are either clustered with hierarchical clustering, or (b) ordered by the number of cell types they are modeled by. Diseases are colored by disease category (immune related: red; cardiothoracic: green; cancer: blue; immune cell cancer: purple), and cell types are colored by innate (orange), and adaptive immune system (dark blue).

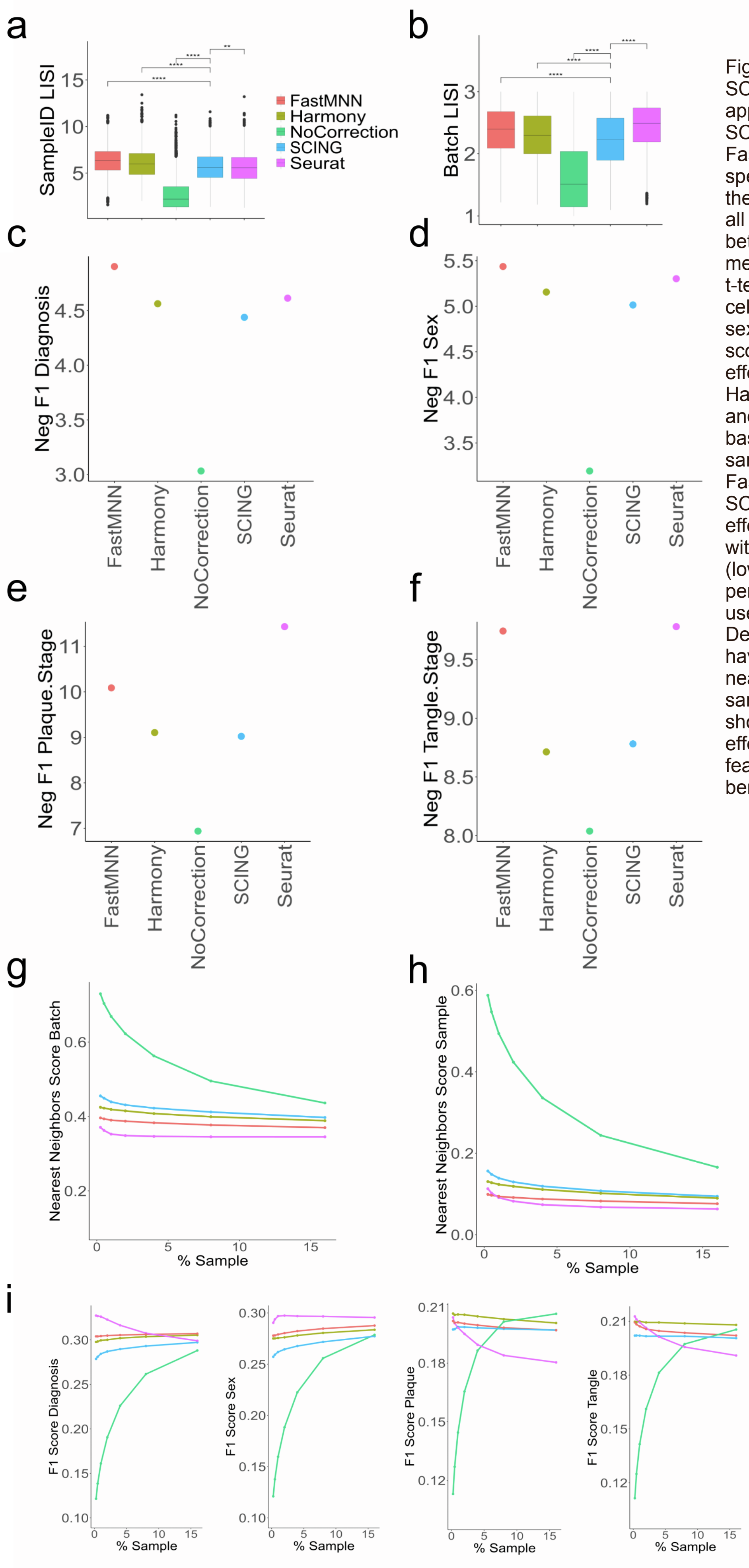

Figure S6. Batch correction effects of SCING compared to other gold standard approaches, related to Figure 5. (a and b) SCING is compared to Harmony, FastMNN, and Seurat at correcting sample specific effects and batch effects based on the local inverse Simpson's index (LISI) for all cells depicted as boxplots. P-values between SCING and each of the other methods were computed with an unpaired t-test. (c and d) SCING is comparable in cell clustering differences in diagnosis and sex in the average F1 score based on LISI scores, while removing sample and batch effects. (e and f) SCING is comparable to Harmony in cell clustering of plaque stage and tangle stage in the average F1 score based on LISI scores, while removing sample and batch effects, however FastMNN and Seurat are better. (g and h) SCING corrects batch and sample specific effects based on the fraction of neighbors with the same batch or sample status (lower is better) for a given cell. This was performed at different fractions of the cells used as neighbors for a given cell. Dedicated batch correction techniques have better batch correction. (i) F1 score of nearest neighbor score with batch and sample effects corrected (higher is better) shows SCING corrects sample and batch effects while retaining biologically relevant features similarly to dedicated benchmarking methods.

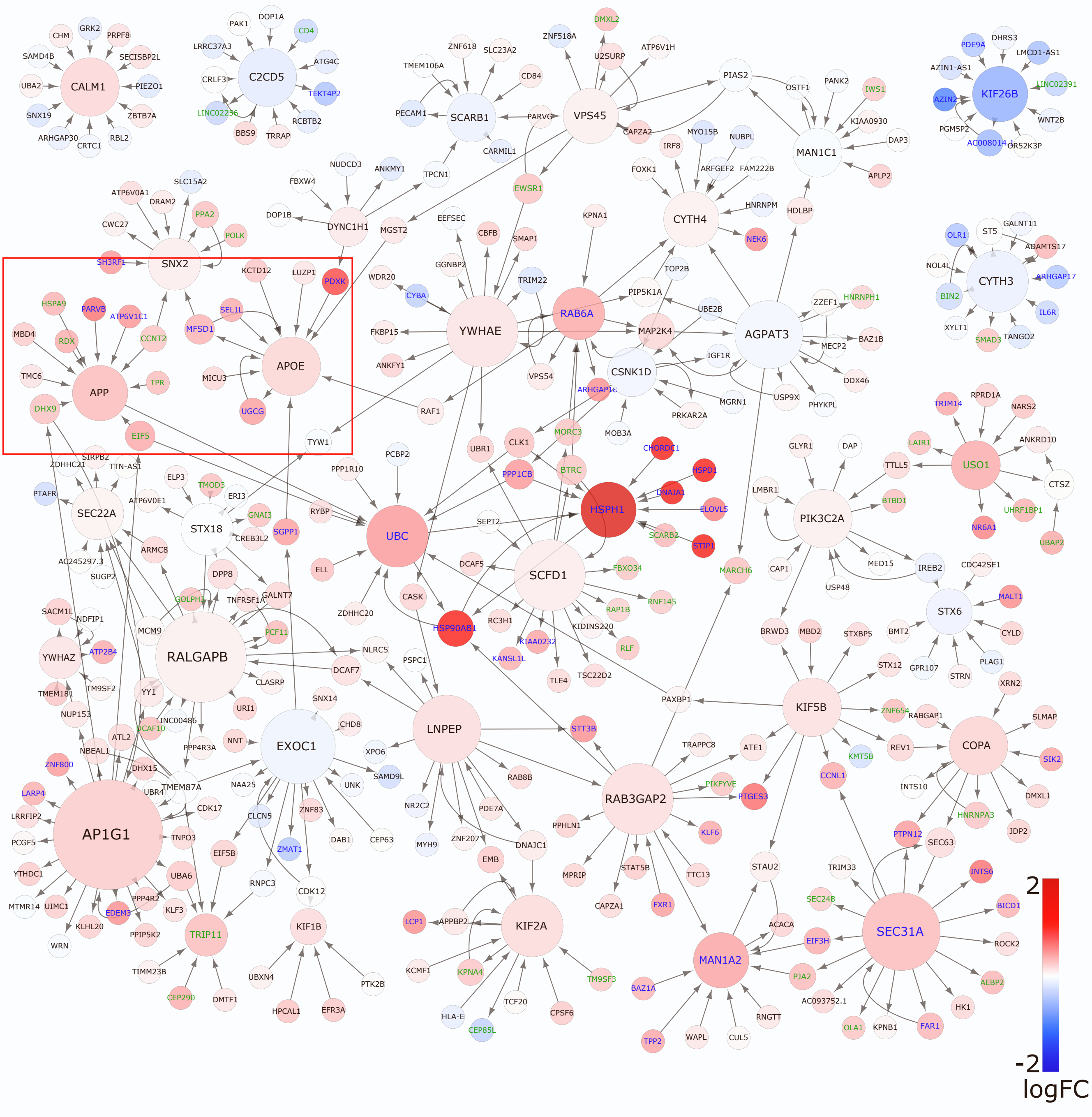

CALM1
CHM
GRK2
PRPF8
SAMD4B
SECISBP2L
UBA2
PIEZO1
SNX19
ZBTB7A
ARHGAP30
CRTC1
RBL2
C2CD5
PAK1
DOP1A
CD4
LRRC37A3
ATG4C
CRLF3
TEKT4P2
LINC02256
BBS9
TRRAP
RCBTB2
SCARB1
ZNF618
SLC23A2
TMEM106A
CD84
PECAM1
PARVG
CARMIL1
VPS45
ZNF518A
DMXL2
ATP6V1H
U2SURP
CAPZA2
PIAS2
OSTF1
PANK2
IWS1
KIAA0930
DAP3
MAN1C1
APLP2
HDLBP
IRF8
MYO15B
NUBPL
FOXK1
ARFGEF2
FAM222B
HNRNPM
CYTH4
NEK6
KIF26B
PDE9A
DHRS3
LMCD1-AS1
AZIN1-AS1
LINC02391
AZIN2
PGM5P2
WNT2B
AC008014.1
OR52K3P
SNX2
SLC15A2
ATP6V0A1
DRAM2
PPA2
CWC27
POLK
SH3RF1
NUDCD3
ANKMY1
FBXW4
TPCN1
DOP1B
DYNC1H1
MGST2
EWSR1
EEFSEC
CBFB
SMAP1
KPNA1
KCTD12
LUZP1
PDXK
HSPA9
PARVB
ATP6V1C1
SEL1L
MBD4
RDX
CCNT2
MFSD1
APOE
TMC6
TPR
MICU3
APP
DHX9
UGCG
EIF5
WDR20
CYBA
GGNBP2
TRIM22
YWHAE
FKBP15
ANKFY1
RAB6A
PIP5K1A
TOP2B
UBE2B
ZZEF1
HNRNPH1
MAP2K4
AGPAT3
BAZ1B
MECP2
IGF1R
CSNK1D
VPS54
MGRN1
USP9X
PHYKPL
DDX46
PRKAR2A
RAF1
TYW1
MOB3A
MORC3
UBR1
CLK1
CYTH3
OLR1
ST5
GALNT11
ADAMTS17
NOL4L
ARHGAP17
BIN2
IL6R
XYLT1
SMAD3
TANGO2
USO1
TRIM14
RPRD1A
NARS2
LAIR1
ANKRD10
TTLL5
CTSZ
NR6A1
UHRF1BP1
UBAP2
HSPH1
CHORDC1
HSPD1
DNAJA1
ELOVL5
SCARB2
STIP1
PPP1CB
BTRC
MARCH6
PIK3C2A
GLYR1
DAP
LMBR1
BTBD1
CAP1
MED15
IREB2
CDC42SE1
USP48
STX6
MALT1
CYLD
BMT2
GPR107
STRN
PLAG1
XRN2
SEC22A
SIRPB2
ZDHHC21
TTN-AS1
ELP3
TMOD3
PTAFR
ATP6V0E1
ERI3
STX18
GNAI3
SGPP1
RYBP
PCBP2
PPP1R10
UBC
SEPT2
CREB3L2
ARMC8
AC245297.3
SUGP2
DPP8
ELL
DCAF5
SCFD1
FBXO34
RAP1B
RNF145
TNFRSF1A
GALNT7
GOLPH3
CASK
ZDHHC20
HSP90AB1
RC3H1
KIDINS220
PCF11
KIAA0232
RLF
KANSL1L
TLE4
TSC22D2
RALGAPB
SACM1L
NDFIP1
MCM9
YWHAZ
ATP2B4
NLRC5
PSPC1
DCAF7
CLASRP
TMEM181
TM9SF2
YY1
NUP153
URI1
LINC00486
SNX14
CHD8
DCAF10
ATL2
NNT
EXOC1
XPO6
LNPEP
STT3B
PAXBP1
KIF5B
ZNF654
RABGAP1
SLMAP
STX12
ZNF800
NBEAL1
PPP4R3A
DHX15
TMEM87A
UBR4
NAA25
UNK
SAMD9L
AP1G1
LARP4
LRRFIP2
CDK17
CLCN5
ZNF83
PCGF5
TNPO3
EIF5B
ZMAT1
DAB1
CEP63
NR2C2
MYH9
PDE7A
ZNF207
DNAJC1
RAB8B
RAB3GAP2
TRAPPC8
ATE1
PIKFYVE
PTGES3
KMT5B
REV1
COPA
SIK2
INTS10
DMXL1
CCNL1
HNRNPA3
JDP2
PTPN12
SEC63
YTHDC1
UBA6
PPHLN1
MPRIP
STAT5B
TTC13
KLF6
STAU2
TRIM33
INTS6
MTMR14
UIMC1
PPP4R2
KLF3
EDEM3
RNPC3
CDK12
EMB
KLHL20
PPIP5K2
TRIP11
KIF1B
WRN
TIMM23B
CEP290
DMTF1
UBXN4
HPCAL1
EFR3A
PTK2B
KIF2A
LCP1
APPBP2
KCMF1
KPNA4
TM9SF3
HLA-E
CEP85L
TCF20
CPSF6
CAPZA1
FXR1
MAN1A2
BAZ1A
ACACA
EIF3H
SEC31A
SEC24B
BICD1
ROCK2
PJA2
AEBP2
AC093752.1
HK1
OLA1
KPNB1
FAR1
TPP2
WAPL
CUL5
RNGTT
2
-2
logFC

Figure S7. Subnetwork of the vesicle-mediated transport pathway from module 2 in the snRNAseq microglia GRN, related to Figure 5. Nodes are colored by their log-fold change (logFC) between AD and Control patients. APOE and APP subnetwork is highlighted (red box).

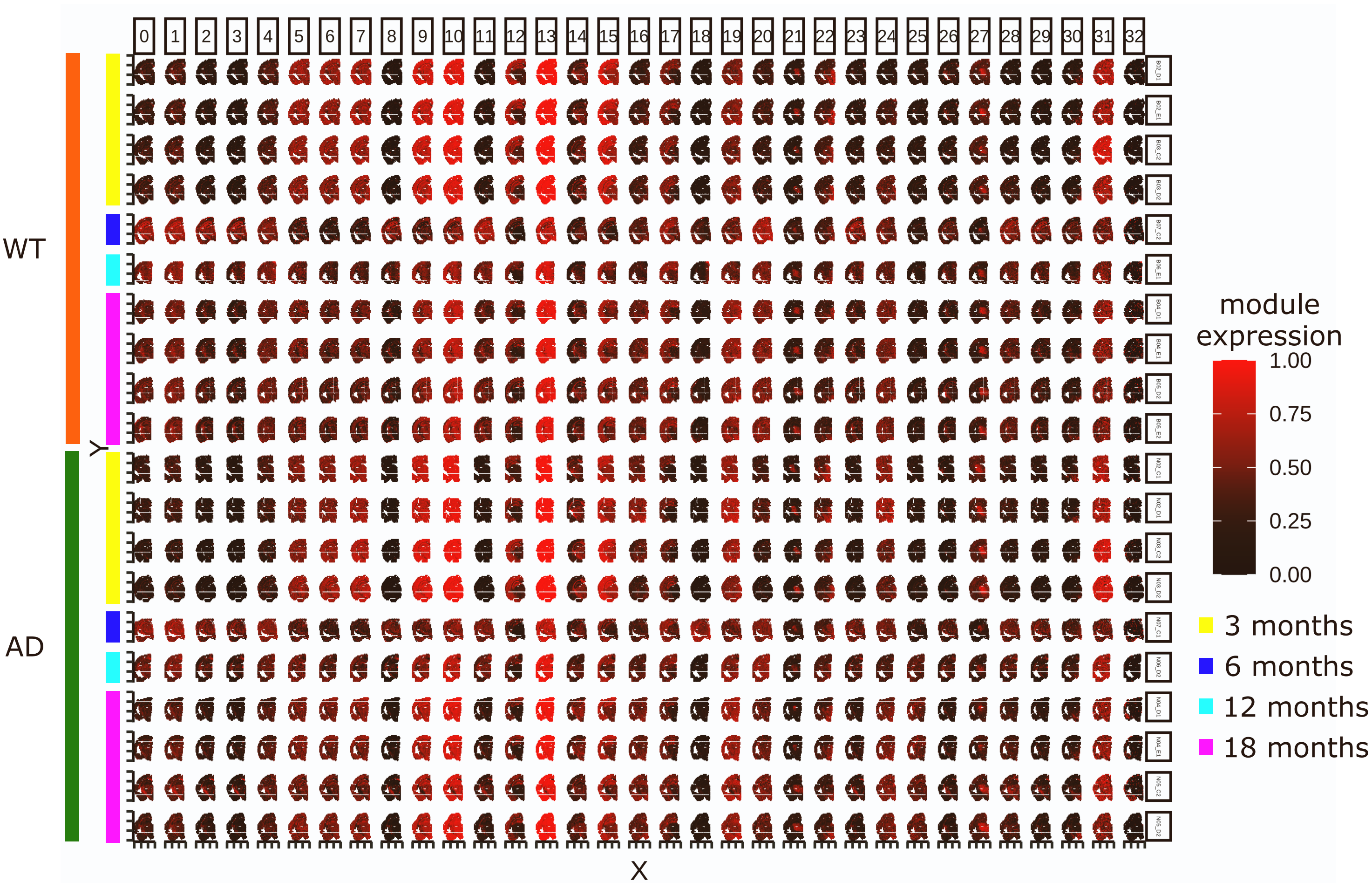

Figure S8. Expression of each module visualized in each brain, related to Figure 6. Mice are ordered by age (3, 6, 12, 18 months) and genotype (WT vs AD) mice.

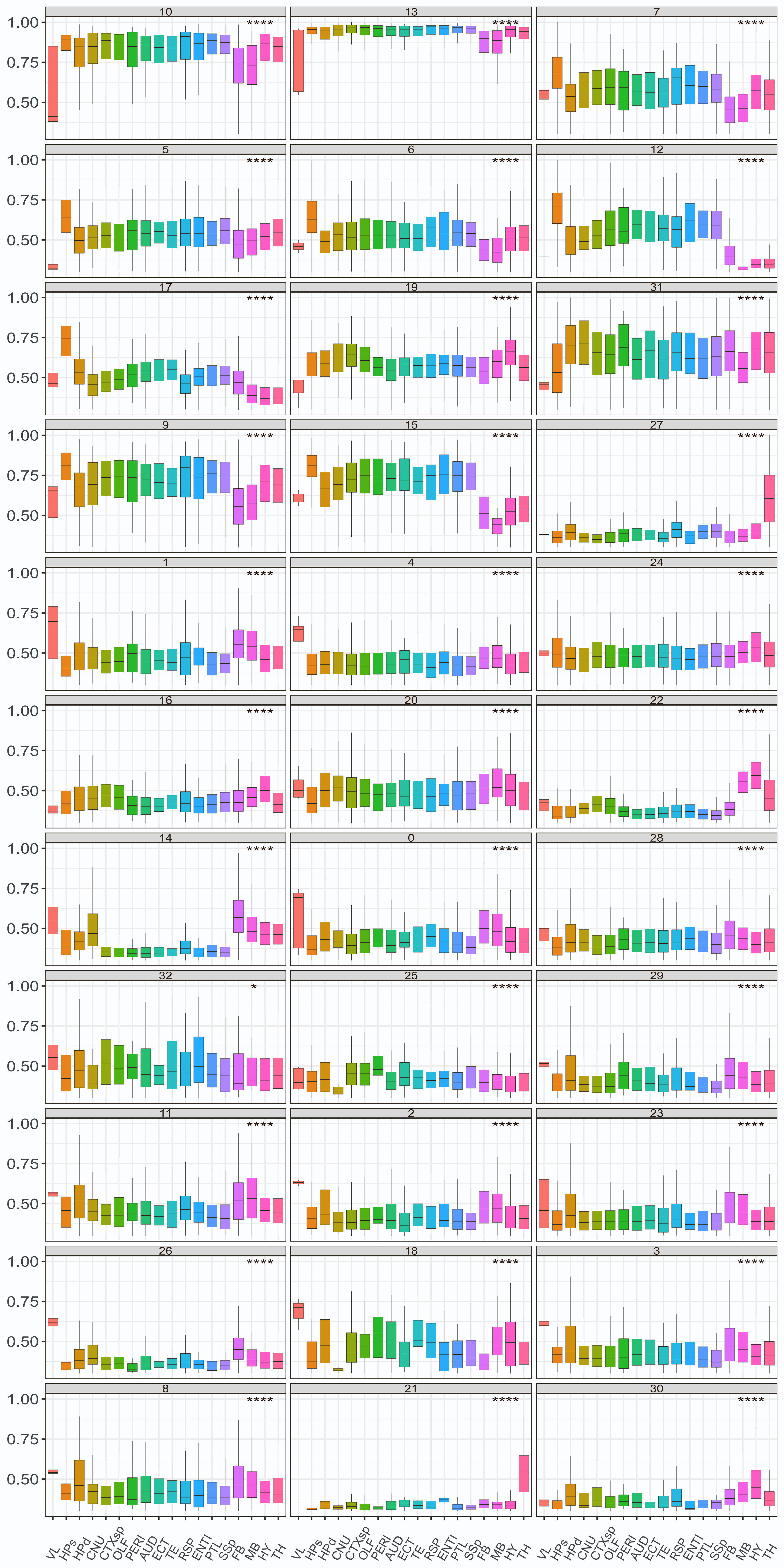
10
13
7
5
6
12
17
19
31
9
15
27
1
4
24
16
20
22
14
0
28
32
25
29
11
2
23
26
18
3
8
21
30
****
*
1.00
0.75
0.50
VL
HPs
HPd
CNU
CTXsp
OLF
PERI
AUD
ECT
TE
RSP
ENTI
PTL
SSp
FB
MB
HY
TH

Figure S9. Distribution of module expression in each region of the brain, related to Figure 6. Significantly variable expression is determined by ANOVA ($p<0.05$).

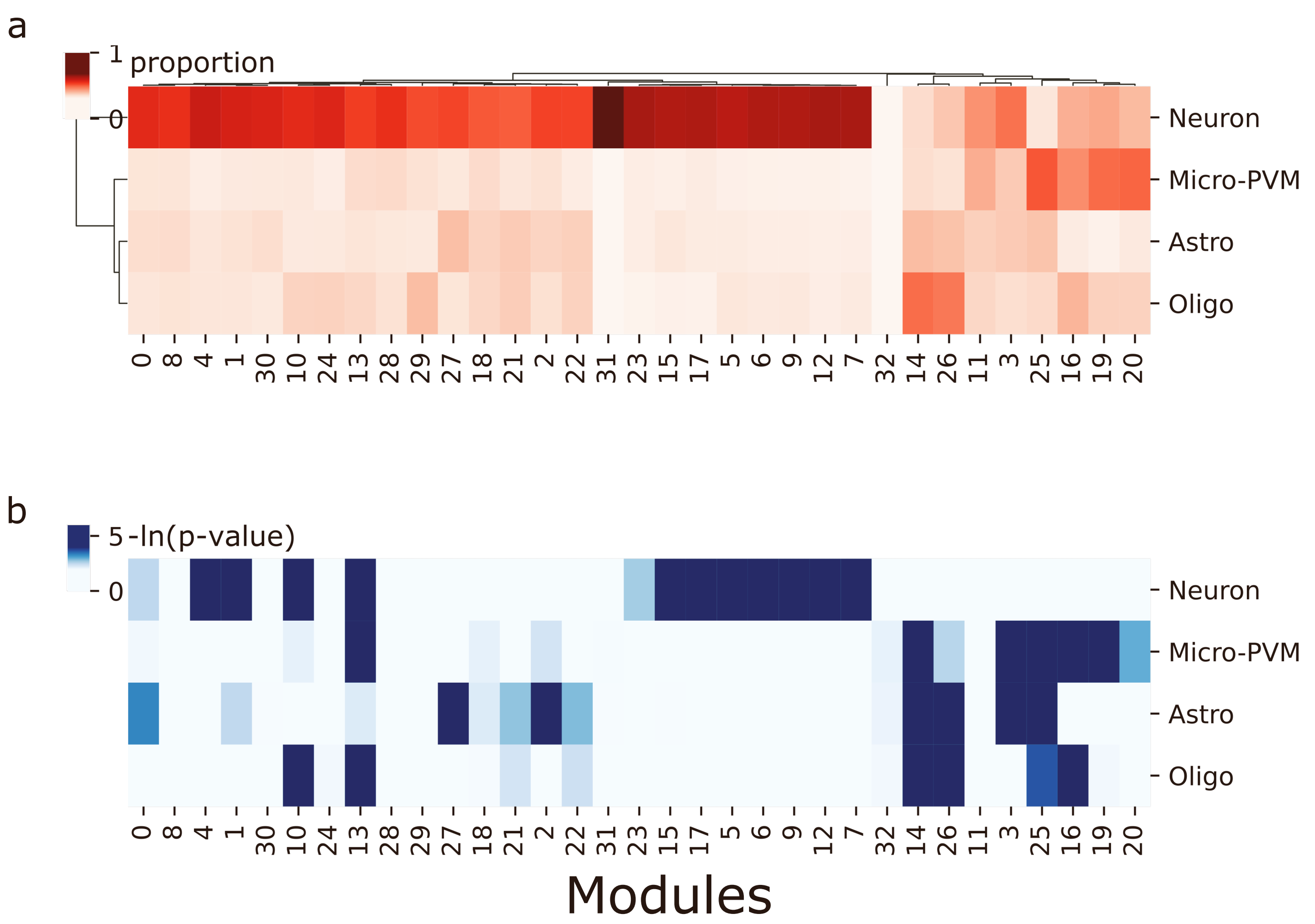

Figure S10. Heat map of cell type proportion and enrichment of marker genes for neurons, microglia, astrocytes, and oligodendrocytes in each module of the Visium whole brain network, related to Figure 6. (a) Proportion of marker genes from each cell type in each module, calculated as (number of cell type marker genes / total genes in module). Many modules mostly have marker genes from neurons, while others have contributions of marker genes from oligodendrocytes (14,16) and microglia (25, 16, 19, 20). (b) Enrichment of marker genes in each module based on Fisher's exact test. Cell type marker genes were determined from the Allen Brain Atlas data.

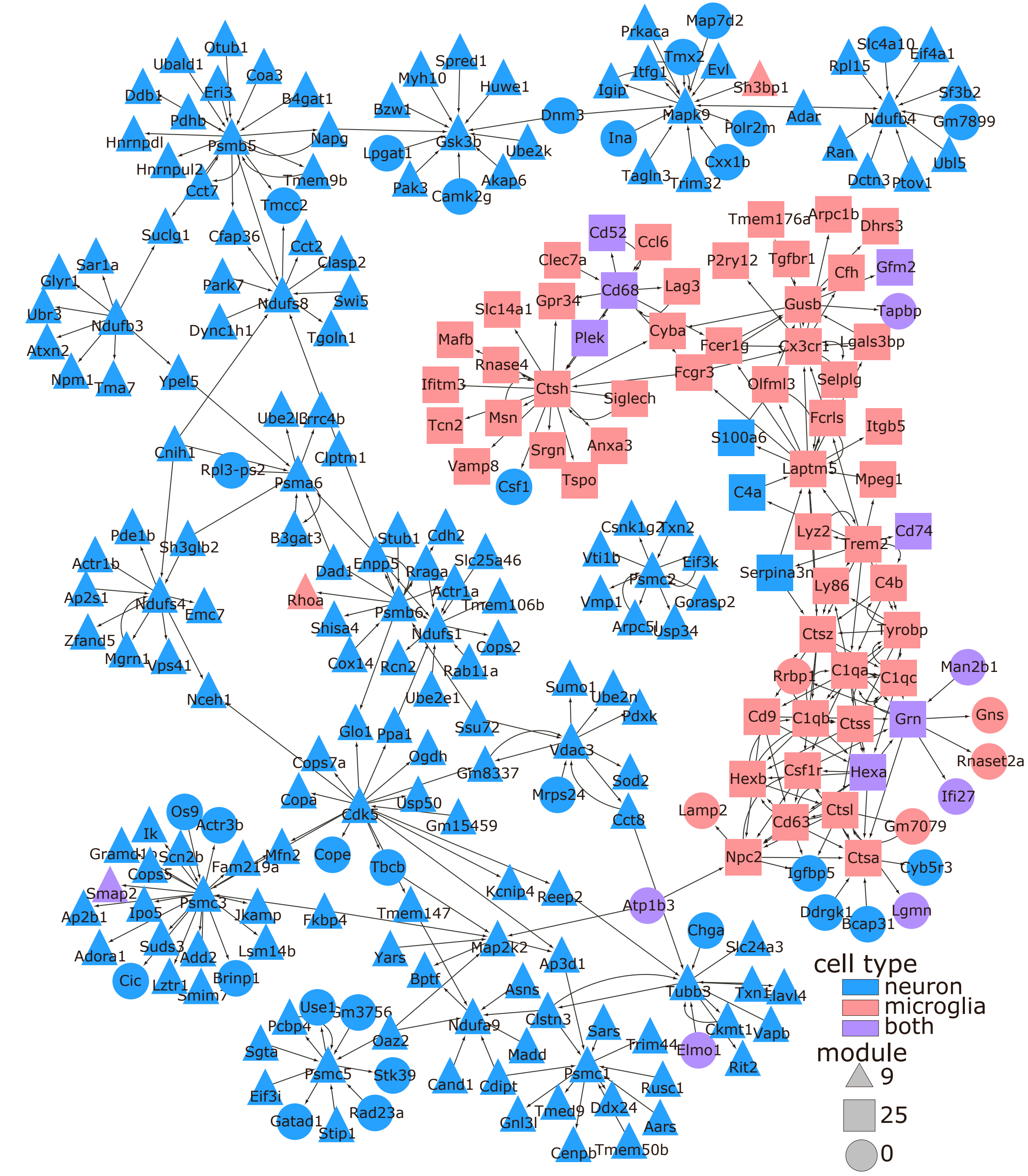
Otub1
Ubald1
Coa3
Eri3
Ddb1
B4gat1
Pdhb
Hnrnpdl
Psmb5
Napg
Hnrnpul2
Tmem9b
Cct7
Tmcc2
Suclg1
Cfap36
Spred1
Myh10
Huwe1
Bzw1
Gsk3b
Dnm3
Lpgat1
Ube2k
Pak3
Akap6
Camk2g
Prkaca
Map7d2
Tmx2
Itfg1
Evl
Sh3bp1
Igip
Mapk9
Ina
Polr2m
Tagln3
Trim32
Cxx1b
Adar
Slc4a10
Rpl15
Eif4a1
Sf3b2
Ndufb4
Gm7899
Ran
Dctn3
Ptov1
Ubl5
Sar1a
Glyr1
Ubr3
Ndufb3
Atxn2
Npm1
Tma7
Ypel5
Cct2
Clasp2
Park7
Ndufs8
Swi5
Dync1h1
Tgoln1
Tmem176a
Arpc1b
Dhrs3
Cd52
Ccl6
Clec7a
Tgfbr1
P2ry12
Cfh
Gfm2
Cd68
Lag3
Gpr34
Slc14a1
Gusb
Tapbp
Plek
Cyba
Mafb
Fcer1g
Cx3cr1
Lgals3bp
Rnase4
Fcgr3
Ifitm3
Ctsh
Olfml3
Selplg
Siglech
Tcn2
Msn
Fcrls
Itgb5
S100a6
Anxa3
Srgn
Vamp8
Laptm5
Mpeg1
Tspo
Csf1
C4a
Ube2l3
Brrc4b
Cnih1
Clptm1
Rpl3-ps2
Psma6
Pde1b
Sh3glb2
B3gat3
Stub1
Cdh2
Actr1b
Dad1
Enpp5
Rraga
Slc25a46
Ap2s1
Ndufs4
Rhoa
Actr1a
Tmem106b
Emc7
Psmb6
Shisa4
Ndufs1
Zfand5
Mgrn1
Vps41
Cox14
Rcn2
Cops2
Rab11a
Nceh1
Ube2e1
Csnk1g2
Txn2
Vti1b
Eif3k
Psmc2
Vmp1
Gorasp2
Arpc5l
Usp34
Lyz2
Trem2
Cd74
Serpina3n
Ly86
C4b
Ctsz
Tyrobp
Rrbp1
C1qa
C1qc
Man2b1
Cd9
C1qb
Ctss
Grn
Gns
Csf1r
Hexb
Hexa
Rnaset2a
Ifi27
Lamp2
Ctsl
Cd63
Gm7079
Npc2
Igfbp5
Ctsa
Cyb5r3
Ddrgk1
Bcap31
Lgmn
Sumo1
Ube2n
Pdxk
Glo1
Ppa1
Ssu72
Vdac3
Cops7a
Ogdh
Gm8337
Sod2
Copa
Usp50
Mrps24
Cdk5
Gm15459
Cct8
Os9
Ik
Actr3b
Gramd1b
Scn2b
Mfn2
Cope
Cops5
Fam219a
Tbcb
Smap2
Psmc3
Ipo5
Jkamp
Ap2b1
Fkbp4
Kcnip4
Reep2
Atp1b3
Tmem147
Suds3
Lsm14b
Adora1
Add2
Chga
Map2k2
Slc24a3
Yars
Ap3d1
Cic
Brinp1
Lztr1
Smim7
Bptf
Asns
Tubb3
Txn1
Elavl4
Use1
Gm3756
Pcbp4
Oaz2
Ndufa9
Clstn3
Ckmt1
Vapb
Sgta
Sars
Trim44
Elmo1
Madd
Psmc5
Stk39
Psmc1
Rit2
Eif3i
Cand1
Cdipt
Rusc1
Rad23a
Tmed9
Ddx24
Gatad1
Stip1
Gnl3l
Aars
Cenpb
Tmem50b
cell type
neuron
microglia
both
module
9
25
0

Figure S11. Subnetwork of module 9 (triangles) and 25 (squares), from the visium AD dataset, related to Figure 6. Marker genes were determined from the Allen Brain Atlas whole brain smart-seq dataset. Nodes are colored by marker gene status (microglia:red; neuronal:blue; both:purple). Cross cell type communication edges are between microglia and neuronal nodes. Module 9 is composed mostly of neuronal genes, while module 25 is composed mostly of mostly microglia genes.

Figure S12. Subnetwork amyloid beta plaque in module 10 from the visium data, related to Figure 6. Nodes are colored by marker gene status (microglia:red; neuronal:blue; both:purple). Hypothesized cross cell type communication edges are between microglia and neuronal nodes. This contains the amyloid-beta plaque stain (colored green).

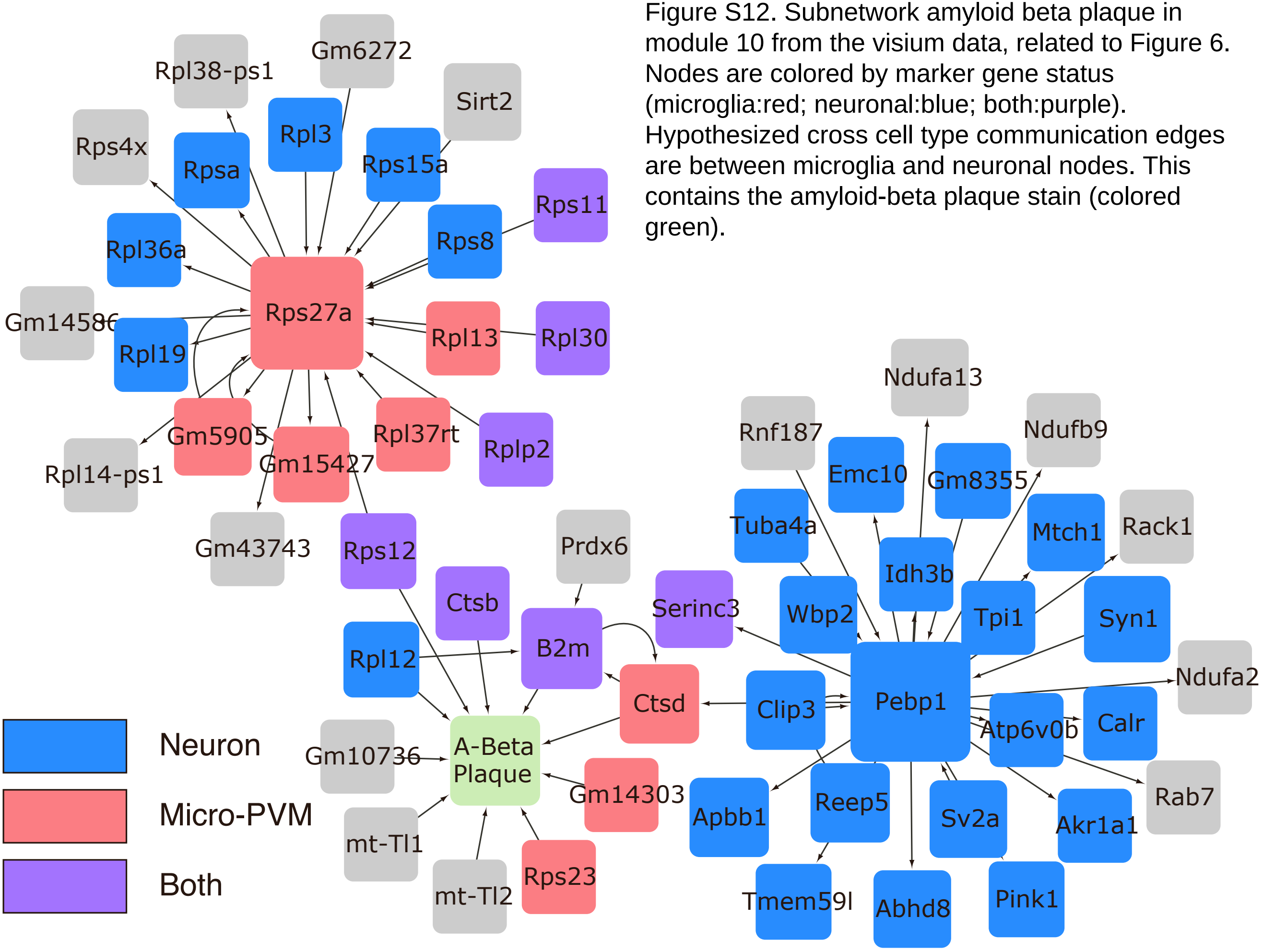

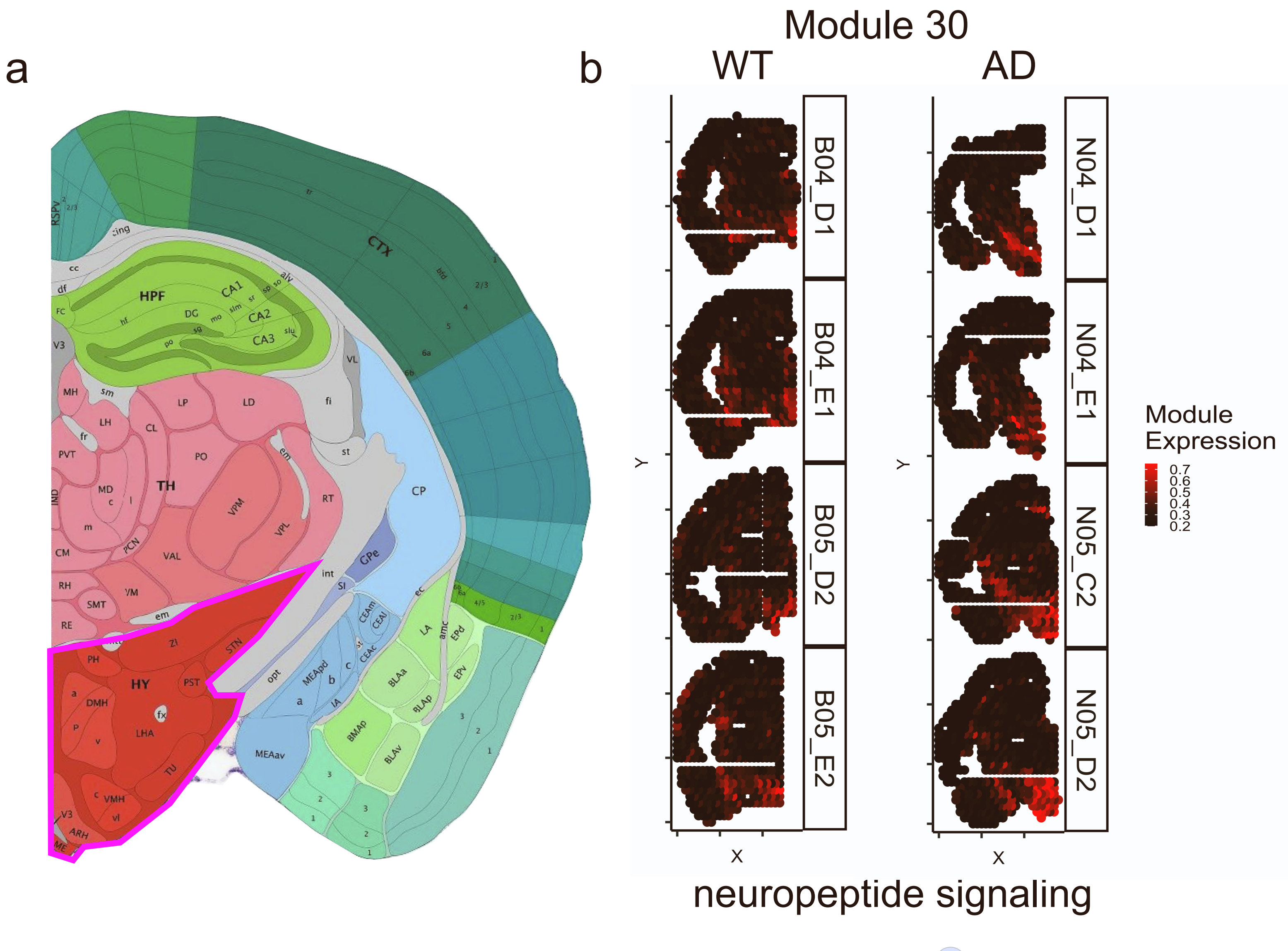

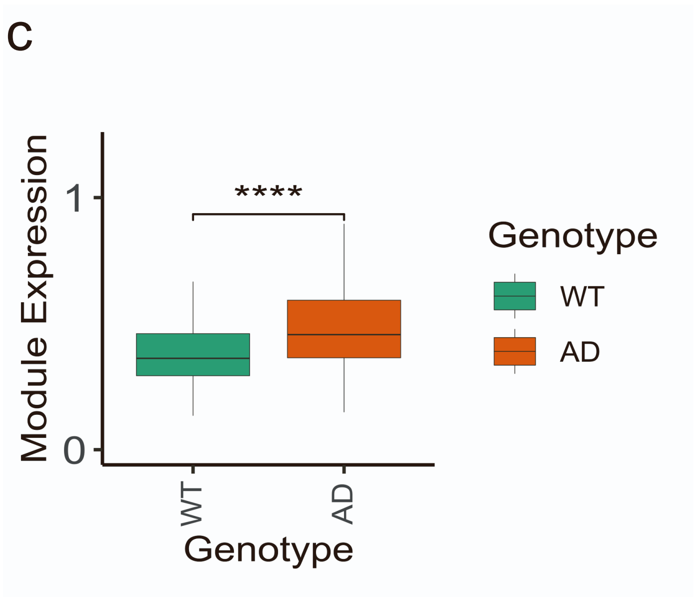

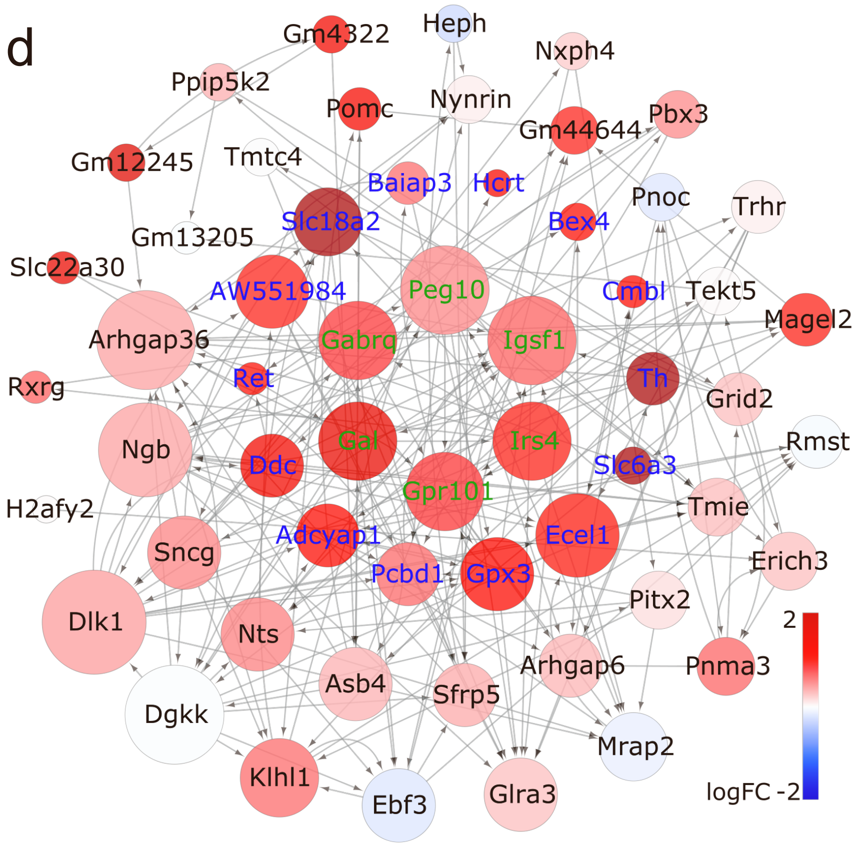

Figure S13. Localization and association of neuropeptide signaling in the hypothalamus, related to Figure 6. (a) Diagram from the Allen Brain Atlas shows the location of the hypothalamus (outlined in pink). (b) Module 30 has localizes to the hypothalamus and (c) has higher expression in AD. (d) The module 30 subnetwork is enriched for neuropeptide signaling. Color of each node represents the logFC in AD vs WT mice. The color of the text represents significance (green: adjusted p-value < 0.05, blue: p-value < 0.01).

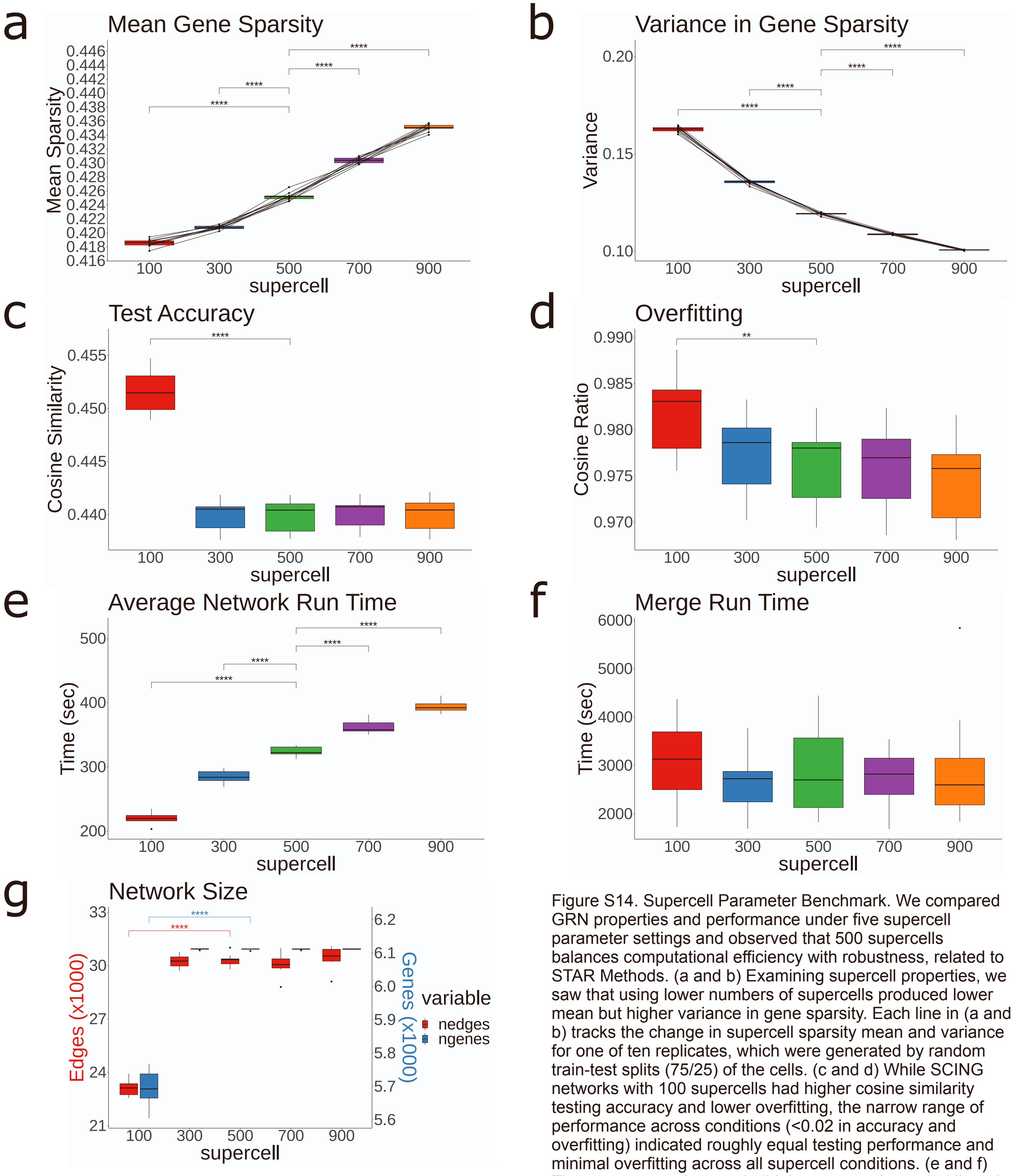

Figure S14. Supercell Parameter Benchmark. We compared GRN properties and performance under five supercell parameter settings and observed that 500 supercells balances computational efficiency with robustness, related to STAR Methods. (a and b) Examining supercell properties, we saw that using lower numbers of supercells produced lower mean but higher variance in gene sparsity. Each line in (a and b) tracks the change in supercell sparsity mean and variance for one of ten replicates, which were generated by random train-test splits (75/25) of the cells. (c and d) While SCING networks with 100 supercells had higher cosine similarity testing accuracy and lower overfitting, the narrow range of performance across conditions (<0.02 in accuracy and overfitting) indicated roughly equal testing performance and minimal overfitting across all supercell conditions. (e and f) The average run times to build these networks scaled linearly with supercell number, and merging run times did not significantly differ from the default 500 supercell setting. (g) The distribution of network sizes increases after 100 supercells and remains consistent for higher settings. The p-values were generated using an unpaired t-test, and significance is shown as (*: $p < 0.05$, **: $p < 0.01$, ***: $p < 0.001$, ****: $p < 0.0001$). Since the purpose of supercells is to control for gene sparsity, we suggest a low mean and variance in supercell gene sparsity as a reference for tuning the parameter, and the user can decide a balance that is best suited for their data.

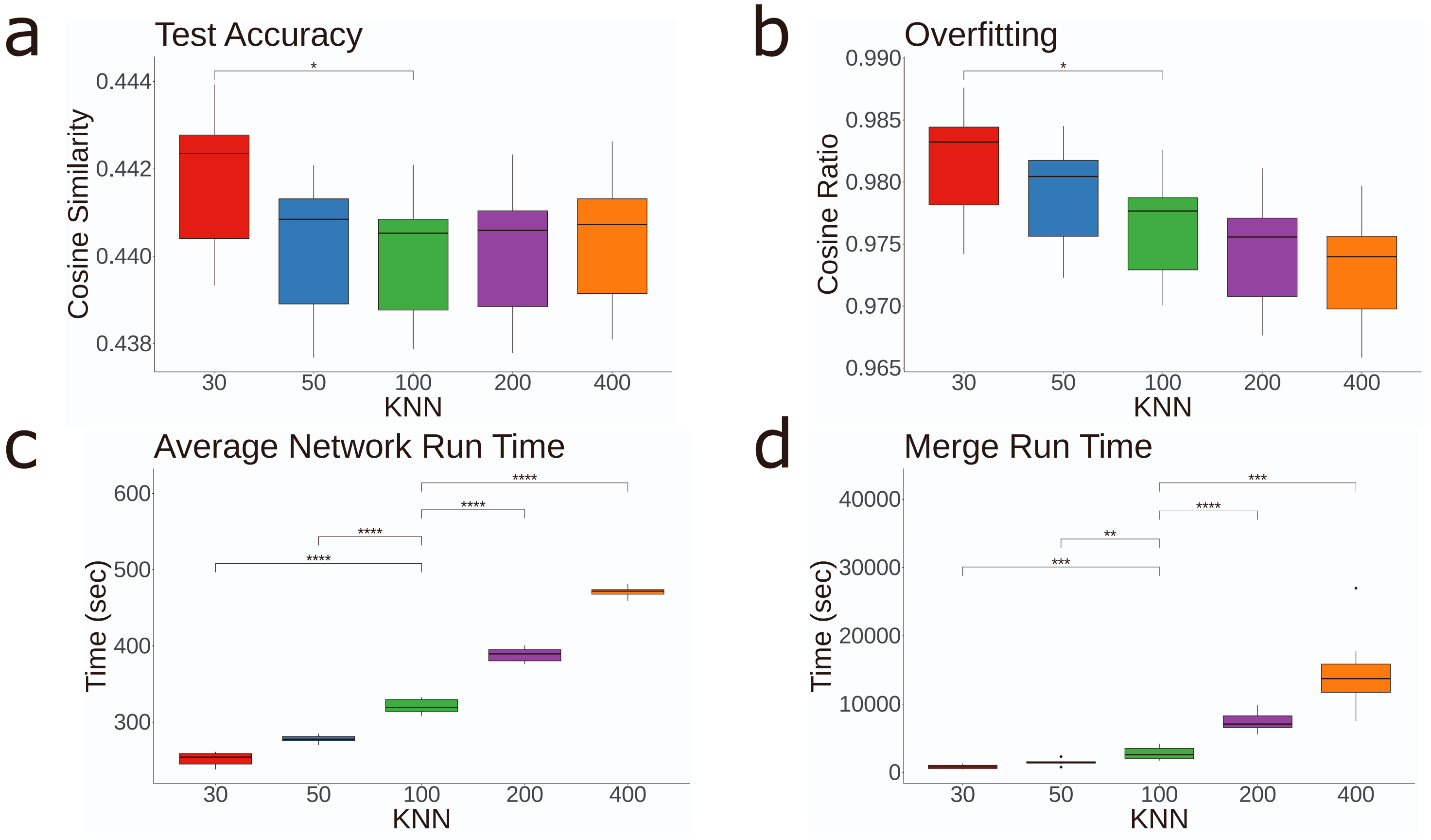

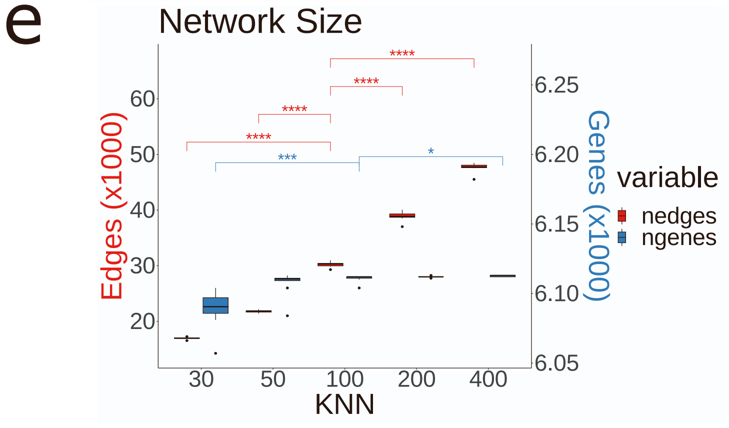

Figure S15. Nearest Neighbor Parameter Benchmark. We compared SCING GRN characteristics across five nearest neighbor gene feature parameters and observed 100 achieves the best balance in computational efficiency and network density in gene expression prediction, related to STAR Methods. (a and b) Gene expression prediction accuracy was distributed around 0.44 regardless of KNN, and the test-to-train ratios ranged between 0.965 and 0.99. (c and d) The subnetwork building run time increased with more nearest neighbors and even more drastically when merging them. (e) Nonetheless, GRNs incorporating more nearest neighbors captured more genes and regulatory relationships in the network. The p-values were generated using an unpaired t-test, and significance is shown as (*: $p < 0.05$, **: $p < 0.01$, ***: $p < 0.001$, ****: $p < 0.0001$). While we chose 100 KNN to mitigate network size, control run times, and comparable performance, this parameter is tunable to fit datasets input by the user.

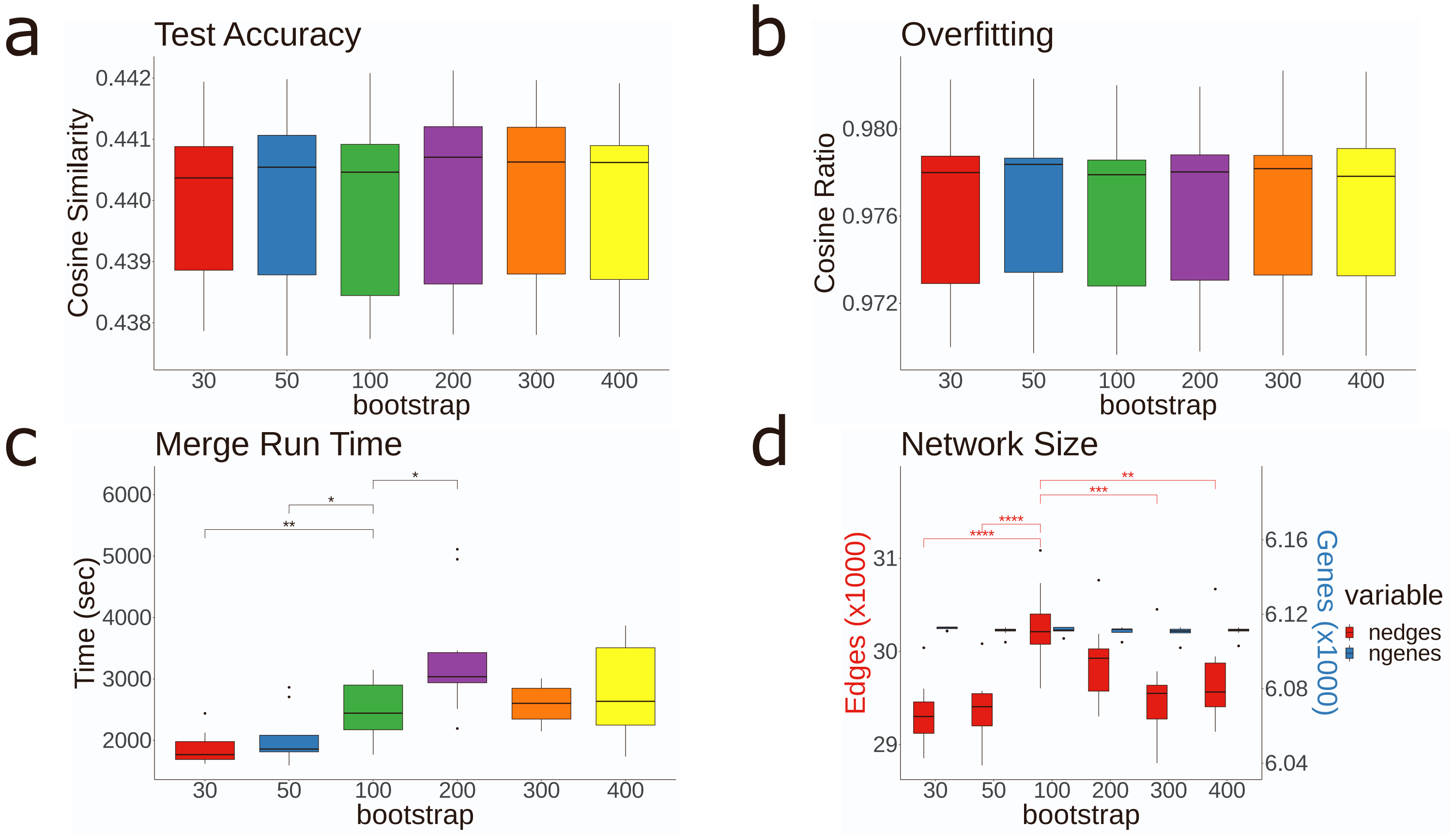

Figure S16. Bootstrap Parameter Benchmark. We tested SCING GRN performance and network properties for six different numbers of subsampled networks and observed integrating 100 subnetworks balances network run time and size, related to STAR Methods. (a and b) Cosine similarities and overfitting in the testing set had negligible changes across bootstrap sizes. (c) GRN merging run times generally increased up to the peak at 200 networks. (d) Using 100 subsampled networks yielded the largest network size across the 10 replicates. The p-values were generated using an unpaired t-test, and significance is shown as (*: $p < 0.05$, **: $p < 0.01$, ***: $p < 0.001$, ****: $p < 0.0001$). While we used 100 subsampled networks in our study given the larger network size, the consistency in accuracy across different bootstrap settings suggests that incorporating above 30 subsampled networks would yield robust GRNs.

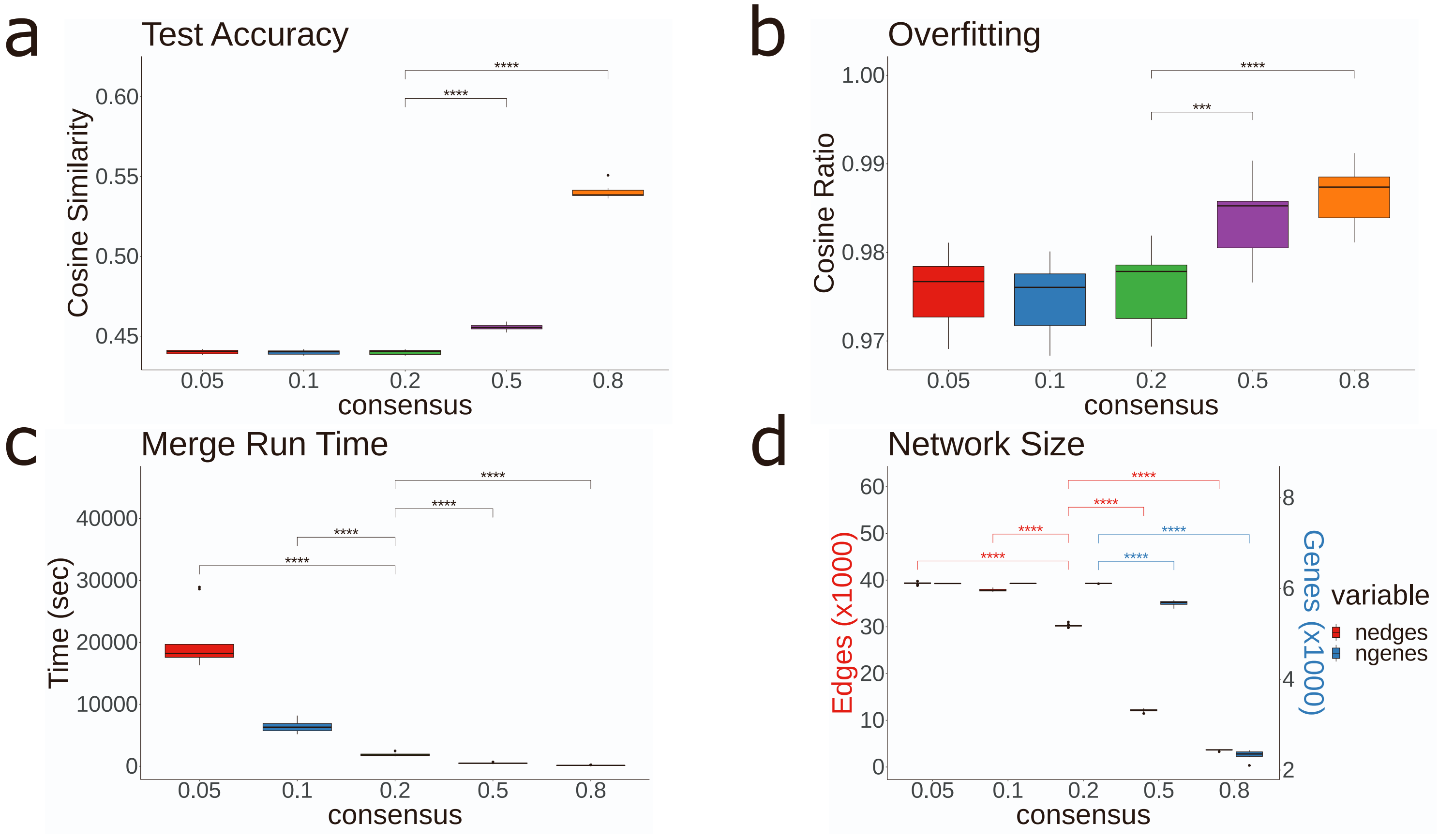

Figure S17: Consensus Parameter Benchmark. We compared GRN characteristics under five consensus parameter settings and observed that a consensus threshold of 0.2 balances computational efficiency with performance while incorporating a large number of genes, related to STAR Methods. (a and b) Gene expression prediction accuracy and robustness were slightly higher for GRNs with more stringent consensus thresholds, but the differences in the scores are narrow. (c) As expected, the runtime to merge subnetworks vastly decreased as the consensus threshold increased. (d) GRNs with higher consensus thresholds resulted in smaller networks in terms of node and edge counts. The p-values were generated using an unpaired t-test, where significance is shown as (*: $p < 0.05$, **: $p < 0.01$, ***: $p < 0.001$, ****: $p < 0.0001$). While a consensus parameter of 0.5 also achieves good performance, we decided 0.2 as the default threshold to include more genes in the network. This parameter is tunable by the user.
